# Delayed diagnosis of Angioimmunoblast T-cell lymphoma presenting with type II Cryoglobulinemia and acute kidney injury: a case report and narrative review of the literature

**DOI:** 10.1186/s12882-020-02125-9

**Published:** 2020-11-07

**Authors:** Xiang-Yang Li, Hai-Yan He, Shu-Ling Yue, Pearl Pai

**Affiliations:** 1 Department of Nephrology, University of Hong Kong - Shenzhen Hospital, Shenzhen, China; 2 Department of Kidney Pathology, Guangzhou KingMed Center for Clinical Laboratory, Guangzhou, China; 3 Department of Medicine, University of Hong Kong - Queen Mary Hospital, Pokfulam Road, Hong Kong, China

**Keywords:** Angioimmunoblastic T cell lymphoma, Dysgammaglobulinemia, Autoantibody, Dysimmunity, Autoimmunity, Monoclonal gammopathy, Cryoglobulinemia, Glomerulonephritis, Cryoglobulinemic syndrome

## Abstract

**Background:**

Angioimmunoblastic T cell lymphoma (AITL) is an infrequent hematological malignancy with variable and often atypical presentations. The presence of dysproteinemia, autoantibodies and systemic involvement in AITL has often led to a delay in diagnosis or even misdiagnosis in practice. We herewith present a case of AITL that primarily presented with acute kidney injury associated with type II Cryoglobulinemia, the underlying cause was only identified 8 months after the emergence of initial symptoms.

**Case presentation:**

A 67-year old woman presented with 2-month history of intermittent joint pain and a 3-day history of bilateral lower limb edema and acute kidney injury. Initial laboratory investigations showed marked hypocomplementemia with positive autoantibodies of ANA, anti-cardiolipin-IgM and direct antiglobulin. The serum and urinary Immunofixation and serum cryoglobulin tests were negative, while the serum free κ to λ light chain ratio was 0.231. A renal biopsy showed a diffuse proliferative glomerulonephritis with intracapillary pseudothrombi formation. There were orderly arranged microtubular structures of 20–35 nm in diameter in the subendothelial and mesangial area on electron microscopy. Shortly afterwards, the patient developed tingling affecting her finger tips and weak hands and legs. A diagnosis of cryoglobulinemia complicated with cryoglobulinemic glomerulonephritis and polyneuropathy was made. She responded well to methylprednisolone, plasma exchange and rituximab. However, 3 months later, she presented with generalized pruritic rash, weight loss, and inguinal lymphadenopathy. A subsequent inguinal excisional lymph node biopsy at month 8 revealed AITL as the underlying disease.

**Conclusions:**

AITL and its associated B cell dysregulation can give rise to autoimmunity and cryoglobulinemia which may conceal itself as the underlying disorder. In various clinical scenarios of auto-immune diseases, it is advisable that the clinicians should take into consideration the multi-faceted lymphoma.

## Background

AITL belongs to the non-Hodgkin’s lymphoma (NHL) and is one of the more common subtypes of peripheral T cell lymphoma (PTCL), accounting for 15 to 20% of PTCL and 1 to 2% of NHL [1]. It is more prevalent in the elderly. The median age of presentation is 62–67 years (range, 20–91), and there is a slight male predominance [2–5]. Cytogenetic study suggests that AITL originates from follicular helper T cell (Tfh). Tfh normally resides in the germinal centers (GCs) within B cell follicles of secondary lymphatic organs [6]. It is known to have derived from naive CD4+ T cell, and plays a crucial role in the GC formation and the modulation of B cell activation, clonal selection, immunoglobulin synthesis, and isotype switch, as well as in somatic hypermutation, based upon cellular interplays between Tfh, B cells and follicular dendritic cells [7]. In normal circumstances, only with the help of Tfh, B cells are able to achieve maturation and transformation into memory B cells and memory plasma cells. Neoplastic expansion of Tfh may result in exaggerated GC reaction, unchecked B cell clonal proliferation, impaired immune tolerance and dysgammaglobulinemia. Consequently, AITL commonly presents with multiple lymphadenopathy and dysproteinemia matching its former descriptions including angioimmunoblastic lymphadenopathy with dysproteinemia (AILD) and immunoblastic lymphadenopathy (IBL) [8]. Its clinical picture may include typical B symptoms of lymphoma such as weight loss, fever and night sweat, with hepatosplenomegaly, elevated lactate dehydrogenase (LDH) level, and features of humoral immunity disturbance and autoimmunity attributable to dysregulatory action of T cells on B cells.

According to reported cases, AITL and its associated conditions may affect the kidney through direct invasion, inducing proliferative or non-proliferative glomerular nephritis (GN), or interstitial nephritis, or vasculitis, or amyloidosis, or cryoglobulinemia [9, 10]. We report here a case of a 67-year-old female with AITL that presented initially with a cryoglobulinemic glomerulonephritis, autoimmunity and polyneuropathy. A delayed diagnosis of AITL was made a few months later. This case demonstrates the importance of considering underlying lymphomatous diseases including AITL in unusual auto-immune diseases and cryoglobulinemia.

## Case presentation

A 67-year old housewife was admitted to the Nephrology Department of our Hospital in December 2018 with 2-month history of intermittent mild to moderate joint pain affecting the right wrist and metacarpophalangeal joints of her right hand, and a 3-day history of bilateral lower limb edema. One month earlier, she underwent a MRI scan in a local hospital for her joint symptoms. The scan showed minor joint fluid with soft tissue swelling of her right wrist and slight degenerative change of her right hand’s carpal bones. Her joint pain improved after some simple analgesia. She reported no rash, and no dry eyes and mouth. There was no parotid gland enlargement or neck swelling either. Ten days before her admission to our hospital, she developed a fever (maximum 38.9 °C) and cough with white sputum. A chest X-ray revealed increased bilateral lung markings. The blood leukocyte count was 7.9 × 10^9^/L (normal 4.0–10.0 × 10^9^/L), hemoglobulin (Hb) 96 g/L (normal 115-150 g/L), CRP level 54 mg/L (normal 0-10 mg/L) and serum creatinine 67 μmol /L (normal 46-92 μmol/L). Her cough and fever responded to a course of cefuroxime given for a suspected lower respiratory tract infection but her legs became increasingly swollen and her serum creatinine rose to 190 μmol/L. She was thereafter admitted to our hospital. Her only past medical history was chronic hypertension that was well-controlled with 5 mg amlodipine daily. She denied any self-medication. There was no significant family history.

At presentation, her temperature was 37 °C, BMI was 21.9 kg/m^2^, and blood pressure was 118/70 mmHg. There was no skin rash or palpable lymphadenopathy. The lungs were clear with no audible heart murmur. The abdominal and neurological examinations were unremarkable. There was bilateral pitting edema from legs to the knees.

Table 1 summarized the results of her laboratory investigations following her admission into our hospital. Her admission full blood count showed a white blood cell (WBC) of 6.82 × 10^9^/L (neutrophils 84%; lymphocytes 12%); red blood cell (RBC) 2.59 × 10^12^/L (3.87–5.11 × 10^12^/L); HB 75 g/L (115–148); and platelet count (PLT) 219 × 10^9^/L (162–341 × 10^9^/L). Her blood film showed a schistocyte < 1%. Urinalysis revealed trace proteinuria; urinary WBC was 13.5/μl (0–23); urinary RBC was 50.6/μl (0–18) and was dysmorphic in appearance. The urinary albumin to creatinine ratio (ACR) and protein to creatinine ratio (PCR) were 153.1 mg/g (< 20) and 261 mg/g (< 200) respectively. Serum CRP was 14.58 mg/L (0–5); and procalcitonin (PCT) level was 1.02 ng/ml (< 0.05). Her serum creatinine was 177 μmol/L (44–80); cystatin C 4.31 mg/L (≤1.03); blood urea nitrogen (BUN) 28.6 mmol/L (2.76–8.07); serum urate 636 μmol/L (142–339); and LDH level was 268 U/L (135–214). The serum alanine aminotransferase (ALT) was 41 IU/L (9–52); aspartate aminotransferase (AST) 37 IU/L (14–36); while serum total protein was 55 g/L (66–87); and albumin 28.9 g/L (35–52). The clotting profile was within normal range.

**Table 1:** Laboratory Data

Variables	On admission	At discharge	Reference range
WBC × 10^9^/L	6.82	9.73	3.89–9.93
Neutrophil %	84.0	82.9	44.0–72.0
LYM %	12.0	8.4	20.0–45.0
RBC × 10^12^/L	2.59	2.47	3.87–5.11
HB g/L	75	74	115–148
PLT × 10^9^/L	219	197	162–341
urinary protein	Trace	Trace	Neg
urinary WBC count/μl	13.5	6.2	0–23
urinary RBC count/μl	50.6 (dysmorphic)	13.2	0–18
ACR (mg/g)	153.1	120.8	<20
PCR (mg/g)	261	464.4	<200
24-h urinary albumin (mg)	61.2	57.7	0–30
24-h urinary protein (mg)	113.69	185	0–140
Procalcitonin (PCT) (ng/ml)	1.02	/	<0.05
Serum CRP (mg/L)	14.58	4.61	0–5
ESR (mm/h)	70	/	0–20
Sodium (mmol/L)	120	141	136–145
Potassium (mmol/L)	4.53	3.45	3.5–5.1
Urea (mmol/L)	28.6	8.0	2.76–8.07
Creatinine μmol/L	177	43	44–80
eGFR (EPI) (mL/min/1.73 m^2^)	25.25	137.75	>90
Bilirubin (μmol/L)	8.1	5.6	0–21
Total protein (g/L)	55	43.6	66–87
Albumin (g/L)	28.9	24.7	35–52
ALT (U/L)	41	18	9–52
AST (U/L)	37	19.7	14–36
GGT (U/L)	38.6	135.2	0–40
ALP (U/L)	84	107	35–105
Calcium (mmol/L)	1.85	/	2.15–2.55
RF (IU/mL)	9.5	/	0–14
ANA	1:320	/	<1:100
ENA	Negative	/	Undetectable
ANCA (U/mL)	Negative	/	0–20
Anti-GBM (U/mL)	Negative	/	Undetectable
β2-GP-1-Ab	Positive	Negative	Undetectable
Anti-cardiolipin-IgG	1.21	1.14	0–12
Anti-cardiolipin-IgM	47.8	3.15	0–12
Coomb’s test	Positive	Negative	Negative
C3 (g/L)	0.26	0.46	0.9–1.8
C4 (g/L)	0.04	0.11	0.1–0.4
IgM (g/L)	4.14	/	0.40–2.30
IgG (g/L)	12.07	/	7–16
IgA (g/L)	1.11	/	0.7–4
Serum IFE	Negative	/	Undetectable
Urine IFE	Negative	/	Undetectable
Free κ light chain (mg/L)	229.25	/	3.30–19.40
Free λ light chain (mg/L)	992.5	/	5.71–26.30
Κ to λ ratio	0.231	/	0.26–1.65
HBV	HBsAg & DNA negative; anti-HBc/anti-HBe negative	/	Undetectable
HCV-Ab	Negative	/	Undetectable
HIV-Ab	Negative	/	Undetectable

Table 1 showed the laboratory investigations of the patient at the time of hospital admission and discharge.

*Abbreviations*: *WBC* White blood cell, *HGB* Hemoglobulin, *PLT* Platelet count, *NEUT* Neutrophil, *LYM* Lymphocyte, *ACR* urinary albumin-creatinine ratio, *PCR* urinary protein-creatinine ratio, *PCT* procalcitonin, *ALP* alkaline phosphatase, *ALT* alanine aminotransferase, *anti-GBM* anti–glomerular basement membrane antibody, *anti-HBc* antibodies to hepatitis B core antigen, *AST* aspartate aminotransferase, *C3* complement C3, *C4* complement C4, *dsDNA* double-stranded DNA, *eGFR* estimated glomerular filtration rate, *ENA* extractable nuclear antigens, *IFE* immunofixation electrophoresis, *GGT* γ-glutamyl transferase, *HBsAg* hepatitis B surface antigen, *HBV* hepatitis B virus, *HCV* hepatitis C virus, *HIV* human immunodeficiency virus, *IgM* immunoglobulin M, *RF* rheumatoid factor, “/” denotes not available

Her serum immunoglobulin (Ig) M was elevated at 4.14 g/L (0.4–2.3); IgG 12.07 g/L (7–16); and IgA 1.11 g/L (0.7–4.0). Serum C3 level was 0.26 g/L (0.9–1.8); C4 level was 0.04 g/L (0.1–0.4), anti-nuclear antibody (ANA) was 1:320 (< 1:100); and rheumatoid factor (RF) was 9.5 IU/ml (0–14). The ENAs (extractable nuclear antigens), anti-double strand DNA (anti-dsDNA), antineutrophil cytoplasmic antibodies (ANCAs) and anti-glomerular basement antibody (anti-GBM) were all negative. The anti-cardiolipin-IgM was 47.8 U/ml (0–12). Anti-β1 glycoprotein and direct antiglobulin test (Coomb’s test) were positive. Anti-cardiolipin-IgG and lupus anticoagulant were negative. Both serum and urinary immunofixation were negative. The serum free κ and λ light chain levels were 229.25 mg/L (3.3–19.4) and 992.5 mg/L (5.71–26.3) respectively with a free κ to λ ratio of 0.231 (0.26–1.65 with normal kidney function, 0.37–3.1 with kidney dysfunction). Serum ferritin was 523.6 mg/L (11–306.8); tumor markers were negative. There was no evidence of past or present viral (V) hepatitis (H) infection with negative anti-HCV, HBsAg, anti-HBsAb, HBeAg, anti-HBeAb, anti-HBcAb. Human immunodeficiency virus (HIV) antibody, syphilis TP-EIA (treponemal-specific enzyme immunoassay), Cytomegalovirus (CMV)-DNA and Epstein-Barr (EBV)-DNA studies were all negative. The first serum cryoglobulin (blood sample obtained on day 12) was negative. Bone marrow biopsy performed on day 5 showed hypercellular marrow with erythropoietic stagnation. The flow cytometry study of a second bone marrow biopsy performed on day 33 did not reveal any phenotypic abnormalities associated with myeloma, lymphoma, leukemia or high risk myelodysplastic syndrome.

A chest and abdominal plain computed tomography (CT) scan taken earlier on day 2 showed patchy exudation along the bronchi and pulmonary blood vessels with small bilateral pleural effusion and a few small to medium size lymph nodes alongside the aortic arch, consistent with the picture of interstitial pneumonitis. There were no ascites or hepatosplenomegaly and no significant lymphadenopathy in the mediastinal, retroperitoneal or inguinal regions. Ultrasound scan showed normal appearance of both kidneys.

**Fig. 1:**
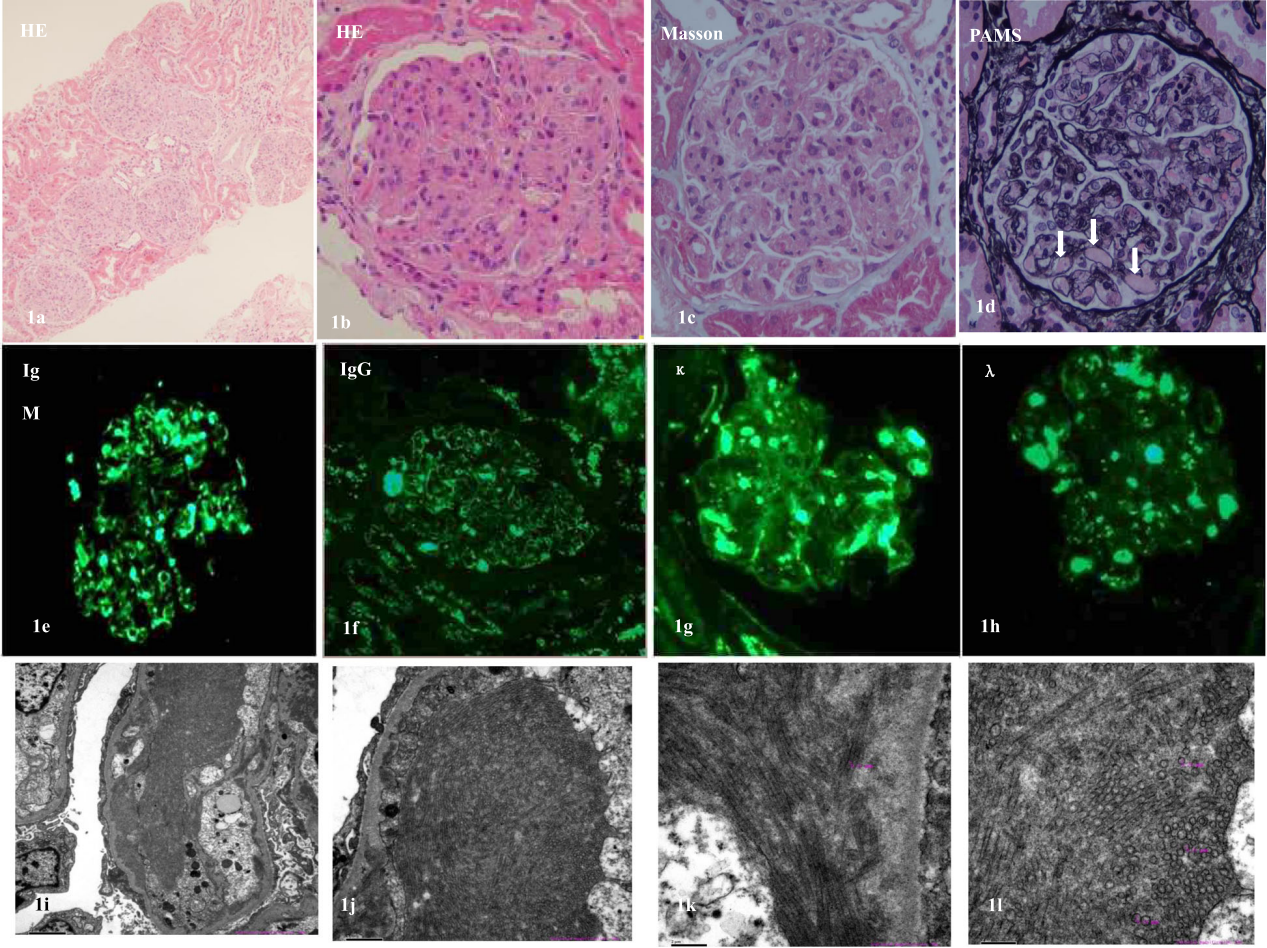
Renal biopsy images. **a**-**c** Light microscopy showing significant endothelial and mesangial hypercellularity (Hematoxylin & Eosin [H&E] and Masson stains; 1a × 100, 1b-c × 400). **d** Light microscopy showing multiple intracapillary hyaline pseudothrombi formation (arrows) but no significant GBM duplication or thickening (Periodic Acid Methenamine [PAM] Silver stain, × 400). **e**-**h** Immunofluorescence microscopy indicates granular deposition of IgM 2+, IgG 1+, κ light chain 3+ and λ light chain 1+, located within the intracapillary thrombi and in the capillary walls (× 400). (i-l) Micrographs of electron microscopy showing orderly arranged microtubular structures of 20–35 nm in diameter within the subendothelial and mesangial areas (× 5000)

The patient was initially treated with intravenous (i.v.) amoxicillin clavulanate for a suspected respiratory tract infection and diuretics for her leg oedema. However, her serum creatinine rose further to 222 μmol/L and a renal biopsy was performed on day 9. The renal histopathology (Fig. 1) revealed a DPGN with intracapillary pseudothrombi formation. The light microscopy (LM) showed that 1 out of 17 glomeruli was sclerosed. There were marked endothelial and mesangial hypercellularity in the remaining 16 glomeruli (Figs. 1a-d). Congo-red stain was negative. There was no thickening or duplication of the glomerular basement membrane (GBM), and no significant epithelial proliferation or crescent formation (Figs. 1b-d). Notably, there was sporadic intraluminal hyaline thrombi formation within the glomerular tufts (Fig. 1d). There was only sporadic focal interstitial inflammatory cell infiltrate in the glomerular and tubular areas. There were signs of mild acute tubular injury, mild tubular atrophy, and about 10% interstitial sclerosis. Arteriolar wall thickening was seen occasionally. Paraffin immunofluorescence (IF) revealed granular deposition (on a 0 to 4+ scale) of 2+ IgM, 1+ IgG, 1+ C1q, 3+ κ light chain and 1+ λ light chain located within the intracapillary thrombi and in the capillary walls (Figs. 1e-h). IgG subclass levels of IgG1–4 were all negative (probably due to a weak IgG positivity of only 1+ by paraffin embedded specimen, and IgG1–4 subclass intensity dispersion). Under electron microscopy (EM), capillary lobulation, localized endothelial proliferation and epithelial vacuolar degeneration could be seen. The GBM measured 400–700 nm in thickness. There were mesangial cellular and matrix expansion and segmental mesangial interposition, with diffuse podocyte foot process effacement. Orderly arranged microtubular structures of 20–35 nm in diameter were seen in the subendothelial and mesangial area (Figs. 1i-l). The extent and degree of mesangial proliferation and interposition, and capillary wall double contouring were only insignificant or minor. Overall features considered, the final pathological diagnosis was considered as DPGN.

**Fig. 2:**
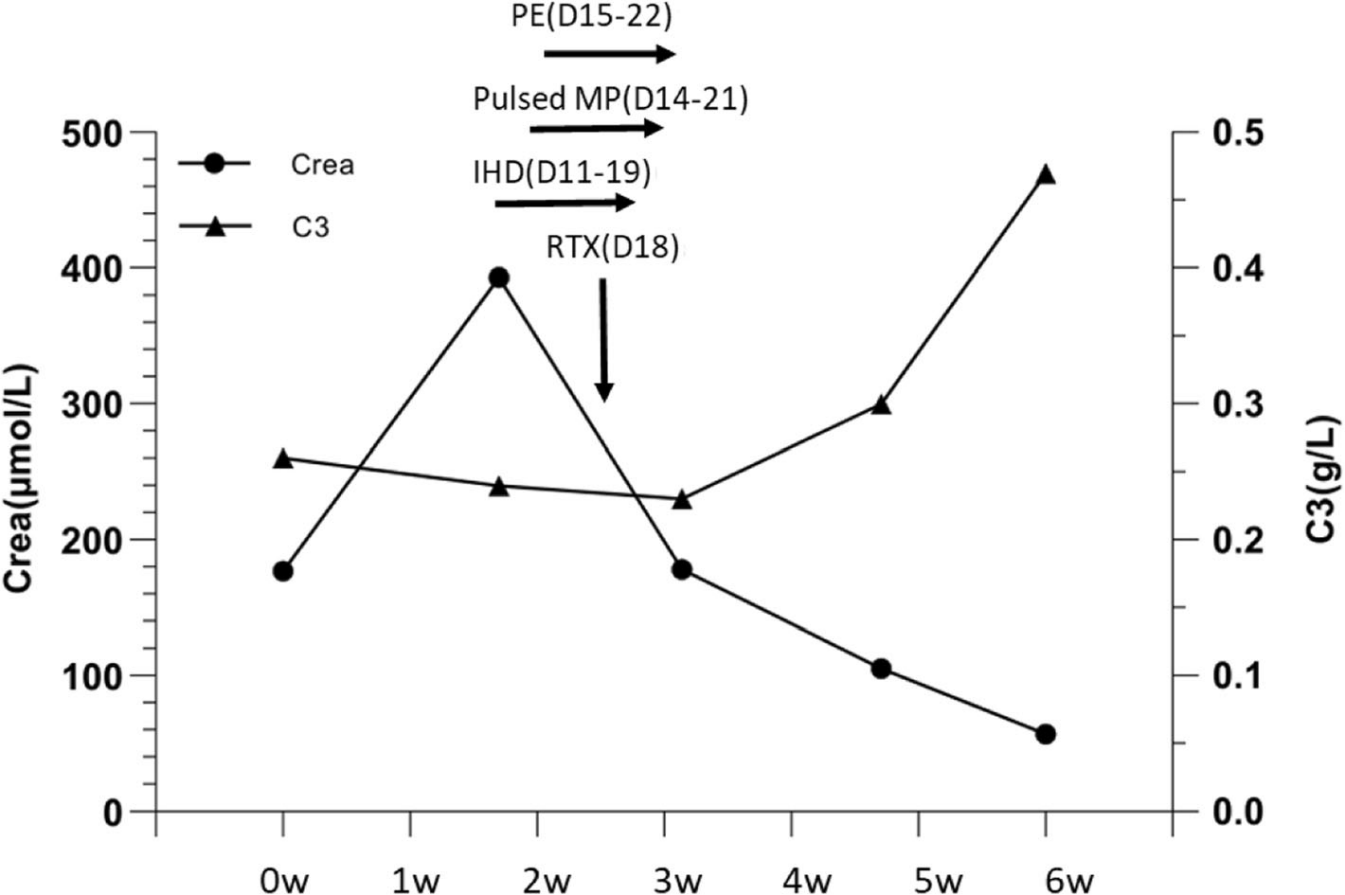
The figure showed the changes of serum creatinine (Crea) and complement C3 during the first 6 weeks (w) in relation to the therapies and the day (D) they were given: Intermittent hemodialysis (IHD); pulsed methylprednisolone (MP); plasma exchange (PE); and rituximab (RTX)

After 10 days of antibiotics and supportive therapy, the patient became increasingly oliguric with fluid overload. On day 11, acute intermittent hemodialysis (IHD) was initiated via a right internal jugular vein temporary dialysis catheter as the serum creatinine rose to 341 μmol/L. On day 13, the patient complained about symmetrical tingling finger-tips and weak hands and legs. A sensory nerve and compound muscle action potential study was carried out which confirmed peripheral polyneuropathy. Meanwhile, her renal biopsy report indicated possibly cryoglobulinemic glomerulonephritis. As she had only recently recovered from a pulmonary infection, she was treated with i.v. methylprednisolone (MP) cautiously from day 14 to day 21 bringing the total treatment dose for this period to 1500 mg. Thereafter, the MP was reduced and maintained at 40 mg daily. She was also given cotrimoxazole 480 mg daily as a prophylaxis against *Pneumocystis jirovecii. On* day 15, she was started on a course of 5 sessions of PE therapy (3.0 L of equal volume fresh frozen plasma and 4% albumin solution, thrice-weekly). On day 18, she was given 600 mg rituximab (375 mg/m^2^). The patient reported rapid improvement of her numbness and weakness after the steroid and PE. The IHD was discontinued on day 19. However, the patient’s cough and sputum returned and the CRP level rose once more. Her serum CMV-DNA became positive with 7.23 × 10^2^ copies/ml and bronchoalveolar lavage fluid (BALF) culture yielded *Candida albicans*. She was treated with ganciclovir and voriconazole. Her chest imaging showed improvement but worsened again on day 29. The i.v. MP was reduced further to 20 mg daily and she was put onto meropenem and voriconazole. On day 33, she was given i.v. immunoglobulin G (IVIG, 0.4 g/kg) for 3 days, after which her lungs and overall condition improved. Able to self-care, she was finally discharged home on day 53, despite a slight tingling in her finger tips. At the time of her discharge, her serum creatinine was 43 μmol/L and the 24 h urinary proteinuria and albuminuria were 185 mg and 57.7 mg respectively. Figure 2 showed the changes of her serum creatinine and C3 complement during her disease course. The discharged diagnosis was acute kidney injury due to mixed cryoglobulinemia, cryoglobulinemic diffuse proliferative glomerulonephritis, underlying monoclonal gammopathy, and interstitial pneumonitis. For personal reasons, the patient declined the suggestions of further MRI or positron emission tomography (PET)-CT scan to explore the cause of the monoclonal gammopathy.

The patient reported being well in the first two months post-discharge and her MP was gradually tapered to 8 mg daily. At two months of discharge, her serum creatinine level was 68 μmol/L; serum C3 was 0.65 g/L; peripheral WBC 8.03 × 10^9^/L; PLT count 273 × 10^9^/L; and Hb 103 g/L. However, at month 3, she reported a generalized pruritic rash, polyarthralgia, weight loss, and groin lymphadenopathy. In June 2019, eight months after the onset of her joint pain, a PET-CT scan was finally taken and which showed extensive lymphadenopathy. An inguinal excisional lymph node biopsy was performed. The pathology reported diffuse effacement of nodal architecture with extensive infiltration of atypical lymphoid cells intermingled with proliferation of small blood vessels. The immunohistochemical study revealed CD3 (+), CD5 (+), CD20 (+), CD21 (+), CD23 (+), CD38 (+), CD10 (+), CD13 (+), PD-1 (+), and BLC-6 (+). The diagnosis was an AITL. The patient agreed to receive one course of CHOP (cyclophosphamide, doxorubicin, vincristine, prednisone) chemotherapy which seemed to have alleviated her symptoms.

## Discussion and conclusion

### Discussion

Lymphoma may affect the kidney tissue via a number of pathomechanisms that include direct invasion, lymphomatous infiltration [11], paraneoplastic syndrome [12] and parenchymal impairment resulted from disease-related dysimmunity, paraproteinemia and cryoglobulinemia [9, 13–15]. It is worthy of note that the pathology of lymphoma-related renal diseases including AITL is multifarious and non-pathognomonic [9–11]. Both glomerular and tubulointerstitial compartments may be infiltrated by lymphomatous cells. If this should be the case, the application of immunophenotypic, cytogenetic or molecular studies on the atypical lymphocytes may be helpful in determining the diagnoses and assist their classification in a far better way than standard morphological studies [13, 16].

AITL was first introduced in 1974 by Frizzera et al. as AILD. The understanding of this disorder has since evolved from a non-malignant condition with neoplastic transformation potential to a hematological neoplasm often associated with aggressive course and unfavorable long-term outcome. The symptoms of AITL are variable and often atypical, and include generalized lymphadenopathy, systemic B symptoms, hepatosplenomegaly, skin rash, polyarthritis, anemia, and pleural effusion/ascites and so on [8, 17, 18]. In our case, the initial complaints were polyarthralgia and anemia which went on to involve the kidneys and the nervous system. Investigations revealed dysproteinemia, significant hypocomplementemia, cryoglobulinemic nephritis and acute kidney injury. The ultimate diagnosis of AITL was made 8 months later. A time lag in the diagnosis of AITL is not uncommon as the early symptoms may be multifarious and nonspecific in nature [8–10]. In a retrospective study of 77 AITL patients, the median time interval between first symptoms and diagnosis of AITL was reported as 3.6 months (1 to 36 months) while the median duration between the initial diagnosis and a final diagnosis of AITL was 2.3 months (0.4 to 29 months) [3].

The disease course of our case illustrated the features of AITL with variable initial presentations and humoral immunity dysfunction associated with dysregulated T-B cell regulation [8]. The anemia might have been related to the positive Coomb’s test but the bone marrow biopsy finding of erythropoietic stagnation and suggestion of pure red cell anemia might have been resulted also from AITL related autoimmune process [19]. Although the serum cryoglobulin was negative in this case, the increased serum IgM, reduced C3 and C4 levels, and renal biopsy findings of endocapillary and mesangial proliferation with intracapillary pseudothrombi, and microtubular ultrastructure of subendothelial electron dense material were all indicative of cryoglobulinemic nephropathy (CN)*.* In fact, we have not been able to demonstrate any atypical lymphocytes in the renal pathology. There was insignificant inflammatory cell infiltrate in the tubulointerstitial or glomerular areas to allow definitive and comprehensive immunophenotypic or cytogenetic studies.

In order to provide a better understanding how AITL may affect the kidneys, we carried out a PubMed search on similar published reports concerning AITL and kidney involvement, using the MeSH terms of “AITL” or “AILD” or “IBL” and “kidney”. As a result of the search, a flowchart of the literature search and a summary of the cases had been compiled, as shown in Table 2 and 3. Hopefully, they might be helpful to show that the types of renal pathologies were variable and comprised an array of glomerular and interstitial lesions.

**Table 2:**
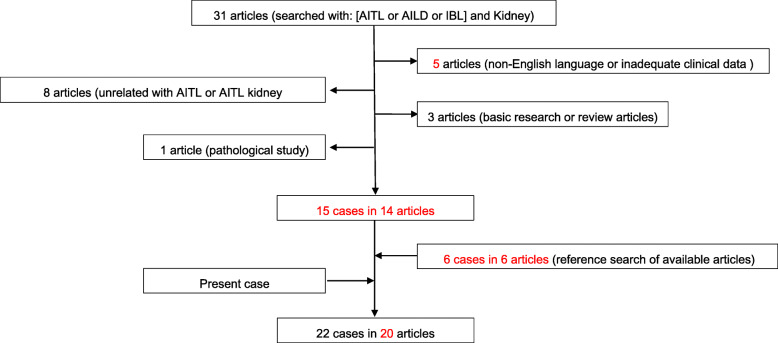
Obtainment of cases and articles.

*Abbreviations*: *AITL* angioimmunoblastic T-cell lymphoma, *AILD* angioimmunoblastic lymphadenopathy with dysproteinemia, *IBL* immunoblastic lymphadenopathy

**Table 3:** Clinical and pathological features of AITL with kidney involvement

No.	Reference	Date of publication	Age	Sex	Hyper-gammaglobulinemia	Monoclonal gammopathy	Serum cryoglobulin	Autoantibody	Manifestations related with kidney involvement	Type of renal lesion/pathological findings	Treatment	Renal outcome
1	Wood [20]	1979	76	M	Yes	N/A	Negative	Coomb’s test	AKI, nephritic syndrome	Minor glomerular change with diffuse podocyte effacement and “full house” pattern of Igs and C3/C4 deposition	Glucocorticoid and CTX	Deceased
2	Wood [20]	1979	79	M	Yes	N/A	N/A	Coomb’s test	AKI, nephritic syndrome	Diffuse granular IgM deposition in the glomerulus	Hemodialysis	Deceased
3	Plazer [21]	1981	64	M	Unclear^*^	Unclear^*^	Unclear^*^	Unclear^*^	Kidney failure	Interstitial nephritis	High dose prednisone	Clinical remission
4	Bhat [22]	1981	77	F	Yes	Urinary kappa light chain	Negative	ANA	Kidney dysfunction	Cast nephropathy, minor glomerular change	No specific treatment	Deceased
5	Resegotti [23]	1983	72	F	Yes	N/A	N/A	N/A	Nephritic syndrome, kidney failure	Lymphoplasmacytic infiltration in the kidney	Melphalan, vincristine, prednisone	Deceased
6	Bello [24]	1985	61	M	Yes	N/A	N/A	Coomb’s test	Fanconi syndrome	not performed	Hydrocortisone	Clinical remission
7	Bignon [25]	1986	70	M	Yes	Negative	Negative	Negative	Proteinuria, kidney failure	Lymphoplasmacytic infiltration in the interstitium	N/A	N/A
8	Staszewski [26]	1988	58	M	Yes	Negative	Negative	Negative	NS, normal kidney function	MCD	No specific treatment	Spontaneous remission
9	Yamazaki [27]	1991	72	M	Unclear^**^	Unclear^**^	Unclear^**^	Unclear^**^	kidney failure	EPGN	vincristine, prednisolone	Deceased
10	Nakamoto [28]	1993	40	M	Yes	Negative	Negative	Coomb’s test	Nephritic syndrome, kidney dysfunction	Interstitial nephritis	CTX, prednisone	Complete remission
11	Duwaji [29]	1995	71	M	Yes	Negative	Positive	Cold agglutinin	AKI	Diffuse proliferative GN	Pulse steroid and CTX, then doxorubicin, CTX, etoposide and vincristine	Deceased
12	Lim [30]	1998	35	M	Yes	N/A	Negative	ANA	NS	AA amyloidosis	CTX, pirarubicin, vincristine, and prednisone	Partial remission of NS
13	Hamidou [31]	2001	65	M	Yes	N/A	Negative	C-ANCA	Nephritic syndrome, kidney failure	N/A	CHOP	Deceased
14	Goto [32]	2004	73	M	Yes	N/A	N/A	N/A	Kidney dysfunction	Diffuse parenchymal infiltration	N/A	N/A
15	De Samblanx [33]	2004	67	M	Yes	Urinary kappa light chain	Negative	C-ANCA	NS, kidney dysfunction	Proliferative GN	Pulse MP + CHOP	Partial remission of NS
16	Miura [34]	2006	70	M	Yes	Serum IgMλ	Positive	Negative	AKI, microscopic hematuria	Cryoglobulinemic MPGN,	Pulse MP + CHOP	Partial remission
17	Argov [35]	2009	46	M	Yes	N/A	N/A	N/A	Kidney dysfunction with normal urinalysis	Giant kidneys, diffuse leukocyte infiltration and parenchymal effacement	CTX, doxorubicin, vincristine, and prednisone	Kidney size shrinkage with improved renal function
18	Togashi [36]	2010	21	M	no	N/A	N/A	Coomb’s test	NS with normal kidney function	MN	CHOP	Complete remission
19	Ambrosio [9]	2012	40	M	Yes	N/A	N/A	Negative	Incidental renal mass, normal kidney function	Unilateral kidney infarction, polyarteritis nodosa lesion in the left kidney	Pegylated liposomal doxorubicin, cytarabine, dexamethasone and nephrectomy of the affected kidney	Partial remission
20	Nanno [37]	2013	30	F	Unclear^**^	Unclear^**^	Unclear^**^	Unclear^**^	NS	EPGN	CHOP	Partial remission
21	Harada [38]	2017	79	M	Yes	Serum IgMλ	Positive	ANA, Coomb’s test	Nephritic syndrome, kidney dysfunction	IgA nephropathy	CTX, pirarubicin, vincristine, and prednisone	Complete remission
22	Present case	–	67	F	Yes	Skewed serum κ/λ	Negative	ANA, ACL, Coomb’s test	AKI, nephritic syndrome	DPGN	MP, RTX and PE then CHOP	Initial remission then relapse

*Abbreviations*: * original article written in Germany with English abstract. ** original article written in Japanese with English abstract, *N/A* not available, *AKI* acute kidney injury, *NS* nephrotic syndrome, *MCD* minimal change disease, *GN* glomerular nephritis, *MN* membranous nephropathy, *EPGN* endothelial proliferative glomerular nephritis, *ACL* anti-phospholipid antibody, *DPGN* diffuse proliferative glomerular nephritis, *CTX* cyclophosphamide, *MP* methylprednisolone, *CHOP* cyclophosphamide, doxorubicin, vincristine, prednisone;

Cryoglobulinemia was classified by the Brouet criterion into 3 types, typeI, II, and III subtype. Their incidence respectively accounts for approximately 10–15%, 60–70% and 20–30% of cryoglobulinemic syndrome [39]. Kidney is frequently affected in mixed (Type II and III) cryoglobulinemia, and to a lesser degree in typeIcryoglobulinemia [40, 41]. The clinical spectrum of CN extends from isolated proteinuria and/or hematuria, acute and chronic nephritic syndrome to nephrotic syndrome. Cryoglobulin activates the complement and sparks off glomerular inflammation and subsequent glomerular proliferation. Our patient presented with mild micro-hematuria and proteinuria that progressed rapidly to renal failure. We suspect that the rapid deterioration of her glomerular filtration function was the result of capillary clog as well as a strong inflammatory reaction in the glomeruli. Although membranoproliferative glomerulonephritis (MPGN) is the predominant pathological phenotype accounting for 70 to 90% of all CN cases [39], other pathological types including endocapillary proliferative GN, mesangial proliferative GN, minimal change disease, and focal segmental glomerular sclerosis have been reported also [9, 19, 42], and they might correspond to a lower grade of inflammation and proliferation [43]. Interestingly, cellular phenotypic analysis has revealed that the endocapillary hypercellularity and capillary wall duplication in CN are sometimes the results of monocytic infiltration, rather than actual endothelial proliferation and mesangial cell and matrix interposition [43].

The mesangial proliferation in our biopsy is slight on LM but the presence of pseudothrombi has been shown as a key to the diagnosis of CN. While cryoglobulin may precipitate either sub-endothelially or intra-luminally in the glomerular tuft [43], the presence of intraluminal pseudothrombi, a hallmark of CN, is only observed in one third [43] to 50% [44] of patients. In our case, the diagnosis of CN was further substantiated by electron microscopy (EM) findings. In cases of CN, electron dense deposits can be found in all glomerular compartments of subendothelial, mesangial, and epimembranous areas. The most characteristic ultrastructure of cryoglobulin-containing deposit on EM has been found to be parallel-arrayed microtubular or curvilinear/annular structure which appears as cylindrical bodies measured 20–30 nm in diameter such as observed in our case. The annular or curvilinear structures are the cross-section of the curved hollow-centered cylinders [10, 43, 44]. Less common ultrastructure phenotypes include long straight parallel fibrils, short poorly oriented fibers in small bundles, and amorphous deposit [44]. The presence of osmiophilic bodies and crystalline inclusions in the endothelial and mesangial cells resulted from the phagocytosis of cryoprecipitate has lent support to the diagnosis of CN [42, 44]. Notably, Immunotactoid GN is characterized by glomerular microtubular structure which may be morphologically indistinguishable from that of CN but without the intracapillary thrombi. Additional clinical data are needed to distinguish the two entities while the presence of cryoglobulinemia and extra-renal involvement favor a CN diagnosis.

Although the detection of serum cryoglobulin supports the diagnosis of cryoglobulinemic disease, failure to detect cryoglobulin does not rule out cryoglobulinemia [45, 46]. Cryoglobulins are temperature sensitive and difficult to be measured. Then the sensitivity of the test is low. For instance, the use of visual inspection of 1% or more cryocrit (considered positive) may negatively impact the test [46]. It is known that the serum level of cryoglobulin of an individual patient may fluctuate during the disease course [43]. Peripheral and end-organ entrapment of cryoglobulins may also result in a false negative. In our patient, any of the factors mentioned might have been contributory to our negative test result for serum cryoglobulin. To resolve these drawbacks, clinicians might turn to immunoelectrophoresis of Igs, histopathological evaluation of affected organs, and correlation of clinical data. In our patient, we deduced the presence of a circulatory monoclonal immunoglobulin (MIg), as indicated by a skewed serum free κ to λ light chain ratio. Since the tests of immunofixation and free light chain assays using serum or urine samples partially overlap each other, test of both should be performed to complement each other and enhance sensitivity [47]. In this case, we might postulate that the negative serum and urine immunofixation (room temperature) might have been resulted from cryoprecipitation of the monoclonal immunoglobulin.

Two bone marrow biopsy studies and a cytometry analysis of our patient have failed to support the diagnosis of a type I cryoglobulinemia, which is generally associated with MGUS or a B-cell lineage malignancy (such as multiple myeloma, Waldenström macroglobulinemia, or chronic lymphocytic leukemia). Type I cryoglobulinemia is typically characterized by the presence of high cryoglobulin concentration, hyperviscosity syndrome and frequent skin lesion. None of these were present in our case. The IF findings of our patient’s kidney biopsy (IgM 2+, IgG1+, C1q 1+, with κ light chain predominance; IgM, IgG, and light chains deposition in the intraluminal and subendothelial space) were suggestive of a mixed cryoglobulinemic nephritis. In our case, the skewed serum free light chain ratio argued against a type III cryoglobulinemia. We would argue strongly that our patient suffered most likely from Type II cryoglobulinemia associated with monoclonal IgMκ and polyclonal IgG. The normal level of rheumatoid factor might have been resulted from end organ/kidney entrapment or consumption of IgM cryoglobulin. In a series of cases reported by Trejo et al. [19], 47/50 (94%) cases of Type II cryoglobulinemia were composed of monoclonal IgMκ and polyclonal IgG. In the same study, the rate of low serum C4 and C3 was 66.7 and 20.3% respectively. The significantly depressed serum C3 and C4 levels in our patient indicated strong classical pathway activation and complement consumption that would have contributed to her AKI.

Due to abnormal T-cell/B-cell interaction and defective antibody production in AITL, dysproteinemia and autoantibodies are common in AITL. About 30–51% of AITL cases manifested hypergammaglobulinemia, and as many as 10–27% of patients demonstrated a detectable monoclonal gammopathy [2, 3]. The dysregulated B-cell proliferation was probably the underlying cause of our patient’s serum free light chain abnormality (κ, 229.25 mg/L; λ 992.5 mg/L; κ/λ ratio of 0.231). The rapid response of our patient’s kidney injury to therapies of pulsed steroid, RTX and PE could be explained by the potent suppression of glomerular inflammation, clearance of IgM-secreting monoclonal B cell clone, and rapid reduction of the circulatory cryoglobulin level. This combined immunosuppressive regimen has also been recommended in the management of other life-threatening or rapidly progressive cryoglobulinemic diseases [48]. Unfortunately, our patient suffered a relapse a few months later. In a small cohort of 25 newly diagnosed patients with AITL, the addition of RTX to CHOP produced good short term response with no benefit on progression-free survival [49]. Like M-H Delfau-Larue et al. [49], we have also found it tempting to speculate that while RTX might have disrupted the T-cell/B-cell interaction and produced short term response, the persistent tumor microenvironment might have brought about a re-emergence of the lymphoma.

Effective and durable disease control of AITL depends on combined chemotherapy and/or autologous stem cell transplantation. CHOP regimen is commonly used as a standard treatment with complete remission rate of around 53% [8]. In spite of active treatment, AITL has a 5-year overall and failure-free survival of 33 and 18% respectively [2]. It looks as if that the long-term treatment of our patient relies on subsequent T-cell targeted consolidation therapies.

## Conclusion

AITL is an infrequent hematological malignancy with variable but nonspecific presentations. AITL associated dysimmunity may lead to multi-organ involvement, while the presence of autoantibodies, cryoglobulinemia and monoclonal gammopathy may conceal AITL as the underlying disorder. A negative test result on serum cryoglobulin does not rule out cryoglobulinemia. Cryoglobulinemic nephropathy is most often associated with MPGN but it may also manifest as other histopathology. Knowledge and awareness of the features of AITL and its pathomechanism may alert clinicians to its early diagnosis and management.

## Data Availability

The authors affirm that all the data supporting our findings are included in the manuscript.

## Abbreviations

AITL: Angioimmunoblastic T cell lymphoma
AILD: Angioimmunoblastic lymphadenopathy with dysproteinemia
CN: Cryoglobulinemic nephropathy
DPGN: Diffuse proliferative glomerulonephritis
GC: Germinal center
IHD: Intermittent hemodialysis
MP: Methylprednisolone
MPGN: Membranoproliferative glomerulonephritis
NHL: Non-Hodgkin’s lymphoma
PE: Plasma exchange
PTCL: Peripheral T cell lymphoma
RTX: Rituximab
Tfh: Helper T cell
